# Let’s Do Engineering: Engineers and Creative Practitioners Experiences of Co-creating Activities and Resources for 3–7 Year-Olds, and Teacher Evaluation of Resource Effectiveness

**DOI:** 10.1007/s10643-025-01858-2

**Published:** 2025-03-01

**Authors:** Helen Bridle, Rebecca Donnelly, Annie Padwick, Gnanathusharan Rajendran, Joe Shimwell, Carol Davenport

**Affiliations:** 1 Institute of Biological Chemistry, Biophysics and Bioengineering, Heriot-Watt University, Riccarton, Edinburgh, EH14 4AS UK; 2 NUSTEM, Faculty of Engineering and Environment, Northumbria University at Newcastle, Newcastle, NE1 8ST UK; 3 Department of Psychology, School of Social Sciences, Heriot-Watt University, Edinburgh, EH14 4AS UK

**Keywords:** Engineering, STEM, Early years, Teacher perspectives, Curriculum evaluation

## Abstract

Let’s do Engineering was a research and engagement project running for 2 years in Scotland, aimed at broadening perceptions of engineering in the early years (ages 3–7). Engineer role models and creative practitioners co-created educational resources which were tested across a variety of settings, including preschool and primary schools. Twenty engineer role models developed over thirty different activities, reaching in excess of 4000 children across Scotland. This article focuses on the impact of the project on the engineer role models, creative practitioners and educators using a mixed methods approach, with data from surveys, interviews and focus groups (n = 49). This study draws out best practices for fostering collaborations between science, technology, engineering, and mathematics (STEM) professionals and creative practitioners (artists) as well as for designing engineering activities for early years audiences. Key evaluation findings were that teachers reported high pupil engagement and valued the diversity of engineering role models, but challenges were identified in linking the resources with child-led early years pedagogies, and the complexity and length of some activities and resources. We have gained knowledge on what educators of 3–7 year olds are looking for when searching for STEM learning materials, and have identified factors that help or hinder the use of learning resources in classrooms.

**Supplementary Information:**

The online version contains supplementary material available at 10.1007/s10643-025-01858-2.

## Introduction

Engineering, in multiple countries, has a well-documented skills shortage, and there is often a lack of gender equality, e.g., in the UK in 2021 just 16.5% of the engineering workforce were female (Engineering UK, 2022). Recent research has focused on how the use of engineering activities and resources in early years education is a critical part of tackling the skills shortage (Pattison & Ramos Montañez, 2022), highlighting that career limiting decisions are taken at a young age (McMahon & Watson, 2022), and can be strongly influenced by stereotypes (Mulvey & Irvin, 2018). Beyond careers, engineering has often been more neglected than other Science, Technology, Engineering, and Maths (STEM) subjects in the curricula, despite a tendency to describe children as “natural engineers” (McClure et al., 2017; Pattison & Ramos Montañez, 2022). A lack of training, support, and confidence for educators is said to play an important role, with 75% of teachers reporting that they lack sufficient knowledge of engineering, and ways to teach engineering (Macdonald et al., 2021).

Let’s do Engineering (the focus of this article) arose from a research and engagement project, and comprises an educational intervention for 3–7 year-old children, based upon linking classroom activities with engineer role models. Involving engineer role models and creative professionals in the design of early years resources is a novel approach. The main goal of Let’s Do Engineering was to highlight the diversity in engineers and engineering topics, along with exploring how a creative approach to teaching could support engineering learning. An additional aim of the project was to educate engineers in the importance of early years engagement. This article evaluates the Let’s do Engineering intervention, addressing the impact on the participants as well as the educator end-users, aiming to highlight important generalisable factors to inform future similar engagement approaches. The generalisable factors presented for the design of future engagements will be of value to early years educators, STEM education researchers, and STEM outreach and engagement professionals.

## Literature Review and Theoretical Framework

### STEM Initiatives at Early Years

DeWitt et al. (2013) have demonstrated that although children (aged 10+) enjoy science in school, they don’t aspire to a science career. Similarly, Padwick et al. (2022) have shown that younger children (aged 5–7) did not relate science learning in school with scientists or science careers and noted that understanding of engineers and engineering is likely to be even more restricted. Early childhood science education research is growing as a field (O’Connor et al., 2021), and STEM initiatives are increasingly targeting younger audiences (Wan et al., 2021; MacDonald et al., 2023). However, since engineering is not well represented within pre-16 school curricula in the UK, it receives less attention in education than the other STEM disciplines, and younger pupils lack opportunities to engage with engineering in their formative years (Edmonds et al., 2022).

Within early years engineering research there have been various main lines of enquiry, including exploring the perceptions and stereotypes of young children in relation to engineers and engineering (Ata-Aktürk & Demircan, 2022; McGuire et al., 2020); as well as studying how children exhibit different engineering behaviours in different conditions, e.g., with different play environments or different pedagogical approaches (Gold et al., 2021; Lippard et al., 2017). A recent review by Master (2021) described how stereotypes at a young age are linked with future STEM interest and engagement, with stereotypes emerging in preschool and strengthening with age. While there is still a lot of work to be done to understand the impact of different stereotypes (e.g., interest, ability, the effect of awareness versus endorsement) the case for addressing stereotypes in early years is clear. With regards to engineering, a recent systematic review of the Draw an Engineer Test revealed that overall children perceive engineers to be males, working individually, outside on manual tasks (Rodrigues-Silva & Alsina, 2023). Younger children seem to have more of a tendency to draw civil structures and workers with tools and machinery, whereas older children have more understanding of the role of design in engineering (Rodrigues-Silva & Alsina, 2023).

Specific educational interventions, designed to introduce young children to engineering have been developed, e.g., Wee Engineer (EiE), If You Were an Engineer What Would You Do? (Welcome to Primary Engineer), Engineering PlayWorld (Fleer, 2021), STEM Person of the Week (Shimwell et al., 2023), family classes like those developed under a design-based research methodology (Ata-Aktürk & Demircan, 2021) or robotics-based activities (Elkin et al., 2016). These interventions have demonstrated success in reducing stereotypes (Shimwell et al., 2023), broadening understandings of engineering (Ata-Aktürk & Demircan, 2021), and more successfully engaging girls (Fleer, 2021). Only the study by Shimwell et al. (2023) specifically utilises role models to support the link between in-setting STEM learning and broader careers.

There are few engineering curricula aimed at early years and so the current study focuses on the description and evaluation of a new intervention combining engineer role models with classroom activity ideas.

### Engineers Designing Curricula: Barriers, Experiences, and Co-creation

Engineers and scientists face multiple challenges in undertaking public engagement activities, including a lack of time, knowledge, skills, and confidence (which is particularly evident for early years engagement), lack of value/support from colleagues and institutions and a lack of funding (Mackay et al., 2020; Woitowich et al., 2022). However, participation in school outreach activities has been shown to build undergraduate science students’ attributes (e.g., communication, teamwork, self-confidence) and incorporate outreach into their views of the role of a scientist (Mackay et al., 2020). Many researchers are motivated to engage and need support to implement community-centred approaches to build relationships and generate two-way engagement with the public (Hawke et al., 2020; Woitowich et al., 2022). Co-creation, defined as collaborating with stakeholders to guide the design process, is a common approach to involve the community in the design and development of engagement activities (Durall Gazulla et al., 2020). For education with young children this approach is beneficial as few scientists/engineers have knowledge or understanding of the early years curriculum, of effective pedagogical approaches, or even age-appropriateness of activities in classroom settings (Bogue et al., 2012). Co-creation has been successfully implemented with children (Carballido et al., 2024; King-Kostelac et al., 2022), with technology enhanced approaches (Siouli et al., 2018), and teachers (Gordon & Cullimore, 2023), resulting in increased learning effectiveness (Gelir, 2022). This article explores the perspectives of engineers, from both academia and industry, on public engagement with young children, measuring self-perceptions of skills, confidence and motivation. Previous work has been more focused on academic and scientific researchers.

### Affordances and Constraints for Early Years Educators in STEM

Science is an important way for young children to learn as it builds on the way in which they develop their knowledge of the world around them (French, 2004). Using STEM as a context allows educators to develop children’s curiosity (Raven & Wenner, 2023), problem-solving abilities (Fusaro & Smith, 2018), science and engineering process skills (Okyay, 2016, Bianchi et al., 2017), and subject content knowledge (Lin et al., 2021). Furthermore, the play-based nature of early years education (McInnes, 2021) fits well with the exploratory nature of STEM (McClure et al., 2017).

A key constraint on the integration of STEM concepts into teaching practice is the low number of early years and primary educators with a background in STEM (STEM Learning, 2023; Royal Society, 2014). Although there is a strong willingness in pre-service primary teachers to teach STEM, their ability and confidence to do so may be lacking (Kurup et al., 2019; O’Neill et al., 2023). This pattern of high willingness but low confidence is also seen in those already teaching (MacDonald et al., 2021; Wan et al., 2021), and can be worse in engineering compared to, for example, maths (Leung, 2023; O’Neill et al., 2023). Other issues include negative beliefs about children’s competencies and abilities to undertake engineering, lack of professional training (on both engineering knowledge and appropriate pedagogies), lack of suitable curriculum resources (Lewis et al., 2021; MacDonald et al., 2023; O’Neill et al., 2023; Van Aalderen-Smeets et al., 2012), and lack of time (Jamil et al., 2018). University-school partnerships offer one route to professional development and enhancing teacher confidence in STEM (Mackay et al., 2020). Teacher confidence and experience is important with one study demonstrating that, in classrooms where educators have less than 5 years experience, fewer examples of Engineering Habits of Mind are exhibited by children (Lippard et al., 2017).

Play-based learning is an effective pedagogy for young children (EEF, 2023). STEAM is focused on the integration of different STEM disciplines together with the Arts, and has been studied for early years education, and presents similar challenges to STEM regarding lack of resources and educator knowledge (Su et al., 2024). Stories have been utilised to motivate problems as described in the above section “STEM Initiatives at Early Years”.  While songs are widely used in early years education, there is currently a knowledge gap about their effectiveness (Hamilton & Murphy, 2023), particularly around the value of using songs and STEM (Na et al., 2014). Wider use of the expressive arts and physical activity together with STEM is also under-explored, despite interesting work on embodied learning (Thomas Jha et al., 2020).

This article investigates the attitudes and barriers for teachers undertaking engineering with young children and focuses on the distinguishing features of activities and resources which support greater uptake and engagement, and whether creative arts approaches are viewed positively.

### Theoretical Framework

We take a constructivist approach to children’s learning and development, which is built upon the work of Piaget (1976). Children’s knowledge is actively constructed, with their learning experiences determining the nature of their reality (Chand, 2024). Teachers and early years educators play an essential role in supporting children’s learning and cognitive development, through scaffolding, drawing attention, and presenting materials that that push the boundaries of children explorations and theory development (Jacobs, 2001). Following this perspective, children will develop understanding of engineers and what they do in the world by engaging in play-based learning resources when this is facilitated and supported by educators.

The development and design of the study was guided by the work of Dierking and Falk (2016) who outline six principles for STEM learning research. Three of the principles were particularly pertinent and used in the planning, design and implementation of the current study. First, the project frames STEM learning research within the needs of society through a recognition of the ways in which engineers are able to contribute to solving environmental and other societal problems, and through introducing educators and children to these contributions. Second, the project links STEM learning to children’s everyday lives, both through the use of a play-based approach, and by using examples and materials readily found in early years settings and homes. Third, the project considered how, and why, to engage educators in learning research, and chose to take a co-creation approach to allow educators to support the design and evaluation of activities developed within the project.

Few schools teach STEM as a discrete subject (Lucas et al., 2014). A child’s introduction to formal science is mediated by their educators. The quality of this introduction will depend on the confidence and background of the educators. Van Aalderen-Smeets et al. (2012) propose a framework for primary teachers’ attitudes towards science, which encompasses cognitive beliefs, affective states, and perceived control. Play-based learning has been shown to be an effective pedagogy for young children improving learning outcomes by approximately 4 months (EEF, 2023) and so the current study used the play-based learning approaches familiar to educators, to support children’s enjoyment of STEM, to challenge their gender beliefs and perceived difficulty of STEM, and to ensure that educators retain the locus of control when using the materials. This approach enabled the study to incorporate several aspects to support educators, which addressed the attitudes described by van Aalderen-Smeets et al. (2012), as shown in Table 1.

**Table 1:** Overview of how the van Aalderen-Smeets framework informed the intervention design

Attitude towards (teaching) science (from van Aalderen-Smeets et al., 2012)	Relevant aspects of Let’s do Engineering
Cognitive beliefs	
Perceived relevance	Resources linked with storybooks and mapped to the curriculum
Perceived difficulty	Resources incorporated educator explanations and step-by-step guides to support
Gender beliefs	Resources specifically developed to challenge stereotypes about who can be an engineer
Affective states	
Enjoyment	Using a play-based pedagogy allows educators to use a familiar approach to teaching STEM, and inclusion of creative practitioners also made the activities attractive and enjoyable
Anxiety	Familiarity with the approaches used reduced the ‘unknown’ nature of the STEM activities, potentially reducing anxiety educators may have felt
Perceived control	
Self-efficacy	Variety of resources enabled selection of activities to meet desired goals and match the interests of the educator and children
Context Dependency	Resources were provided with clear instructions about the preparation required overcoming these potential barriers

### Research Questions

The literature review found few interventions aimed at early years engineering, even fewer of which report their evaluation approaches (Padwick et al., 2016) and none that incorporated expressive arts approaches into learning. There is little evidence to determine how to design interventions, with limited knowledge of what works well in which contexts, although some studies have shown that story-led approaches are successful (Fleer, 2022; Leung, 2023). Incorporation of engineer role models has been shown to have a positive impact for older children (Shimwell et al., 2023), and studies suggest that counter-stereotypical imagery can be beneficial for people of all ages (Aladé et al., 2022; Finnegan et al., 2015; Olsson & Martiny, 2018). Engaging educators in learning research about what works is important (Dierking & Falk, 2016), but has not been a major focus of previous research. Similarly, previous work has not directly involved engineers in the creation of resources. Within this study we sought to evaluate the project from multiple perspectives, addressing the following research questions:

- What was the impact of the project on engineer role models and were they inspired to engage in further public engagement activities? RQ1
- How well did engineer role models and creative practitioners work together? What did they learn and what could be improved for future initiatives? RQ2
- How useful did teachers and nursery practitioners find the resources and activities? RQ3
- Which factors controlled whether particular resources or activities were used in various settings? RQ4
- What can we learn to improve the design of future similar projects and to enhance the development of further early years engineering resources? RQ5

The impact on participating children in terms of their engineering skills, attitudes and perceptions is addressed elsewhere, with one article on the impact on perceptions having been recently published (Bridle & Rajendran, 2024).

## Methods

Let’s Do Engineering was a funded project (Engineering and Physical Science Council Engineering Engagement Champions Fund and the Royal Academy of Engineering Ingenious! award) aiming to engage children aged 3–7 years old in engineering experiences; highlighting the breadth of engineering fields, and the diversity of engineering disciplines and application areas. The design and evaluation methods are described below. This was a mixed methods study, using some quantitative data gathered from surveys, although the majority of the data was generated through qualitative approaches using interviews and focus groups (see Table 2).

**Table 2:** Overview of participants

Participant	Definition	Number	Recruitment	Characteristics	Methods
Engineer role models	People working as professional engineers either in industry, policy or academic; or those studying, engineering	20	Via application. Application form circulated through networks (e.g., STEM Ambassadors, engineering institutes) and via press releases	Selected to maximise diversity in terms of gender, engineering discipline, age, and background	Independent evaluator undertook survey (distributed to all participating engineers) and telephone interviews (5 engineers). See SI for details
Educators (teachers)	Teachers or nursery educators, working in schools or nurseries with 3–7-year-olds. Either qualified or in training	23	Via School’s Outreach Officer and STEM educator networks	Majority female, predominantly white; levels of teaching experience varied	Project lead gathered email and telephone feedback (6 teachers). Independent evaluator undertook 1 telephone interviews and 4 focus groups (total 17 educators). Survey data was used to track demographics and indications of science capital. See SI for details
Creative practitioners (artists)	People working in a creative arts field, e.g.. circus, drama, dance, film, graphic design, music	6	Circulated call through networks. Conducted interviews to select final range of artists	Majority female, predominantly white; all with multiple years of experience, and usually expertise in working with 3–7 year olds (target audience)	Independent evaluator undertook telephone interviews (4 practitioners). See SI for details
Children	Children aged between 3 and 7 years old	~ 4000	Via the educators	Roughly equal gender split, mixed social demographics	Impact on children previously reported (Bridle & Rajendran, 2024)

The table provides details of all the different participants, including the definition of each type of participant, the number of that type of participant, recruitment methods, characteristics and research methods

### Participant Selection and Description

There were three main participant roles within the project: engineer role models, creative practitioners, and educators. This section describes how participants were selected and their roles.

An initial call for engineers to take part in the project was shared with authors’ networks, promoted via engineering institutions and other professional bodies, and advertised via a press release; all interested respondents were asked to complete a short application form which asked for details of age, gender, route into engineering, current job role, and public engagement experience. Twenty engineer role models were selected for participation in the project from a total of 37 applicants. Based on information provided in the applications, engineer role models were chosen to maximise diversity, both in personal characteristics and engineering disciplines. Engineer role models were provided with training in the skills and knowledge required to successfully communicate engineering to the target audience (3–7 year old children) and the project aimed to inspire them to undertake further public engagement work, particularly focused on tackling stereotypes with younger audiences. The main roles of the engineer role models were to:

1. Share their stories about themselves, their work, and how they got into engineering, to provide relatable role-models, which would be presented through various means, e.g., films, images, interview text, posters etc.
2. develop activities and resources representing their area of engineering in ways designed to engage young children (in collaboration with creative practitioners).

In order to explore a range of creative approaches to engineering engagement, creative practitioners were also involved in the project. We define creative practitioners to encompass a broad range of creative and artistic disciplines. Creative practitioners were approached through the research team’s existing contacts and networks, and included film makers, graphic designers, a circus company, a musician, and a dance/drama specialist. The role of the creative practitioners was to support the engineer role models with the creation of resources and activities.

The final participants were the educators, some of which were recruited via the Author’s University School’s Outreach Officer and STEM educator networks. The educators trialled resources in their settings and gave feedback on what worked well along with suggestions for changes and improvements. Further educators were recruited by an external consultant to join a focus group to supplement the evaluation data, ensuring that all activities and resources were reviewed.

The children taking part in the activities were key participants in Let’s Do Engineering although they were not directly involved in the design phase and the current paper focuses on the adult participants presented above. The impact of the intervention on young children’s perceptions of engineers has been published elsewhere (Bridle & Rajendran, 2024).

### Co-creation of Activities and Resources

Based on the literature, Let’s do Engineering adopted an approach of combining engineer role models with play-based activities, using a co-creation method to develop the resources. The project originally planned to bring together engineer role models, creative practitioners and educators during a 2-day workshop to co-create a range of activities and resources for use in schools and nurseries. However, due to the project occurring during the lockdowns associated with Covid-19 pandemic, the co-creation process was shifted to an online event (held in May 2021) incorporating training sessions and break-out discussions between engineer role models and creative practitioners. Educators were not able to attend due to the demands of remote teaching; however, the training did include ex-teachers now working in education research, along with public engagement experts and creative practitioners who had prior expertise of engaging the target audience in formal education settings. The agenda for this workshop included project briefing, training/guidance, introductions to allow everyone to get an idea of the types of work everyone did, and break-out sessions offering the opportunity for small group discussions of initial ideas. The project team facilitated these discussions, dropping into the different break-out rooms to check in on discussions and ensure everyone was given the chance to participate. After the workshop engineer role models and creative practitioners were encouraged to explore collaborations and jointly generate resources and activities, with regular check-ins with education researchers and the project lead. The project lead took an active role in facilitating collaborations and facilitating connections between engineer role models and creative practitioners and suggesting ideas to explore further, particularly where participants were unsure. Ideas were also discussed with teachers leading to adaptation and further development of some of the activities. The activities developed include short engineer role model interviews and films, card games, an activity book, a silent film, circus activities, songs, drama and dance, science experiments, arts and crafts and engineering challenges. Activities were available for testing in schools and nurseries from Autumn 2021, and were free to download and test, with some schools and nurseries allocated specific resources to trial. All activities can be found on the website www.letsdoengineering.com or can be obtained on request by contacting the corresponding author.

### Engineer Role Model Evaluation

Evaluation of this component of the work was undertaken by an external consultant, with the methods developed jointly. Evaluation methods included a survey distributed to all engineer role model participants and a shorter number of telephone interviews designed to explore further how well the co-creation process worked for the engineer role models. See Supplementary Information for details of the questions used.

### Creative Practitioner Evaluation

Creative practitioners attended the workshop and met a range of engineer role models through different break-out room activities. Subsequently, the project lead followed up with the creative practitioners to gather a range of potential ideas and collaborations, encouraging further discussion with the engineers before finalising which collaborations went forward to development. Evaluation of this component of the work was undertaken by an external consultant, through telephone interviews. See Supplementary Information for details of the questions used.

### Activities Evaluation

The resources and activities were shared via contacts and networks of the research team. Printed copies of activity books were delivered free of charge to schools and nurseries, who were encouraged to trial different resources and activities from the selection available on the website. Educators were initially given the freedom to select activities and resources which best suited their setting and needs. However, as testing went on, educators were provided with a suggested selection of activities. The rationale for this change was that the work required to review the 32 options available on the website could be overwhelming and time-consuming. Training and support on each resource and activity were not given; instead, educators were provided with all the instructions and guides (some of which included videos) and given an online overview introduction explaining the types of resource available and key points regarding introducing engineering at early years. Educators were encouraged to utilise a range of resources to expose children to different engineer role models and types of engineering and to feedback on both the resources and the impact on children. Data were gathered from telephone calls and emails with the project lead. See Supplementary Information for details of the questions used.

### Teacher Focus Groups

During Autumn 2022 existing learning from the above methods was consolidated and reviewed with the project team identifying gaps in the evaluation and knowledge generated. Some activities and resources had been utilised more than others but the reasons for this discrepancy were not clear. It was determined that greater teacher feedback would be helpful to better understand the factors which controlled whether educators would be likely to utilise different resources and activities. Such knowledge would be helpful to further develop effective resources and activities and provide useful learning to other science and engineering engagement projects in a development phase. Another external consultant was engaged to undertake this work, utilising a survey and a series of focus groups to explore educator perceptions of Let’s Do Engineering. In total there were four focus groups (with four participants) and one in-depth interview with a total of 17 teachers from across early years (under 5s) and primary school (up to age 8) provision. Prior to the focus groups thirteen of the seventeen participants completed the pre-focus-group survey, which explored their attitudes and perceptions to engineering (questions available on request). The focus group and interview discussions explored what factors influence the resources teachers choose, their experiences of using the website and resources, and how appropriate they found them for their school/teaching. Prior to the focus groups a survey was circulated to the participants, to assess their perceptions of engineering, and thirteen responses were received. See Supplementary Information for details of the questions used.

### Data Analysis

Methodologies were revised over multiple facilitated meetings, using literature and participant expertise to develop a theory of change to support longer-term project aims and identify short-term evaluation goals. From this an iterative approach to question design was undertaken resulting in a final set of surveys and interview questions. Data included survey responses from engineer application forms and evaluation questionnaires (both collected via Microsoft Forms); interviews by one external consultant with engineers, creative practitioners, and teachers on the telephone, (transcribed using otter.ai); email feedback from teachers; focus group teacher surveys and discussions on Teams, which were recorded and scribed. Quantitative survey data was extracted from Microsoft Forms and analysed in Excel. Qualitative responses were analysed taking a grounded theory approach (Charmaz & Thornberg, 2020), with the interview transcripts processed using Dedoose (https://www.dedoose.com/), going through the text line by line noting common themes. Data analysis for the focus group followed the reflexive thematic analysis methodology outlined by Braun and Clarke (2022) and included five steps: familiarisation with the data, generation of initial in vivo codes, generation of codes into initial themes, reflection and re-organisation of these, and subsequent finalisation of these themes. Both these sets of analysis were conducted by external consultants aiming to minimise bias in two ways; educator reporting bias if talking directly to the programme designers and author bias towards programme success. Once themes were obtained the authors combined the findings, refining the ideas and emphasising themes emerging from both sets of analyses.

## Results

### Impact on Engineer Role Models (RQ1)

Eleven engineer role models gave feedback using the survey questionnaire tool and 8 completed all of the survey questions. All engineer role models had attended an introductory workshop and taken part in short meetings. Drop-in feedback sessions were less well accessed (4 respondents). Engineer role models agreed that developing materials for this age group felt challenging, but all agreed that it also felt achievable which indicates that the project was providing an appropriate level of professional development.

“I found the workshop and short meetings were really useful to generate ideas for potential activity and refine those to be pitched at the right level of complexity. It might have been helpful to have more follow-up engagement directly with creative partners developing the activity, or other examples of materials at similar levels or contexts to what we were aiming for, though of course one of the great parts of the project was that we were developing material to fill the present gap.” Engineer 1

Engineer role models reported the project had the greatest impact in teaching them how to design resources and activities for 3–7 year olds, overcoming one of the issues found in the literature, a lack of knowledge limiting outreach (Bogue et al., 2012; Mackay et al., 2020). All were convinced of the value of engaging with this age group, with 90% reporting the project had positively changed their approach to public engagement. The project had inspired the engineer role models that were interviewed to consider their own work in the field of public engagement, and they had become more involved in the STEM public engagement work of their own universities. The project had also impacted some of the engineer role model’s work with undergraduates and they had begun encouraging students to see if they could communicate their project descriptions in a way appropriate for primary school children. This provides evidence that the academic engineers could see the potential benefits of outreach for their university students (Mackay et al., 2020).

### Learning from the Creative Practitioners (RQ2)

Creative practitioners all came with teaching experience with this age group and were all excited and inspired by the engineer role models they worked with. They felt the process could have been improved with a clear ‘contract’ between the engineer role models and creative practitioners, with timescales and budgets made more explicit. All creative practitioners expressed a regret that there was not more time to get to know the engineers, network, and collaborate further.

“There’s so much in common with engineering, which is the same as with circus, you can figure out what’s not working and you can fix it … and the process of figuring it out is really satisfying. Things, when you don’t understand them seem really magic and impossible. And then when you understand them, you can do the impossible. That’s such an amazing thing to transform everything else in your life!” Creative practitioner 1

### Effectiveness of the Resources and Activities (RQ3)

**Fig. 1:**
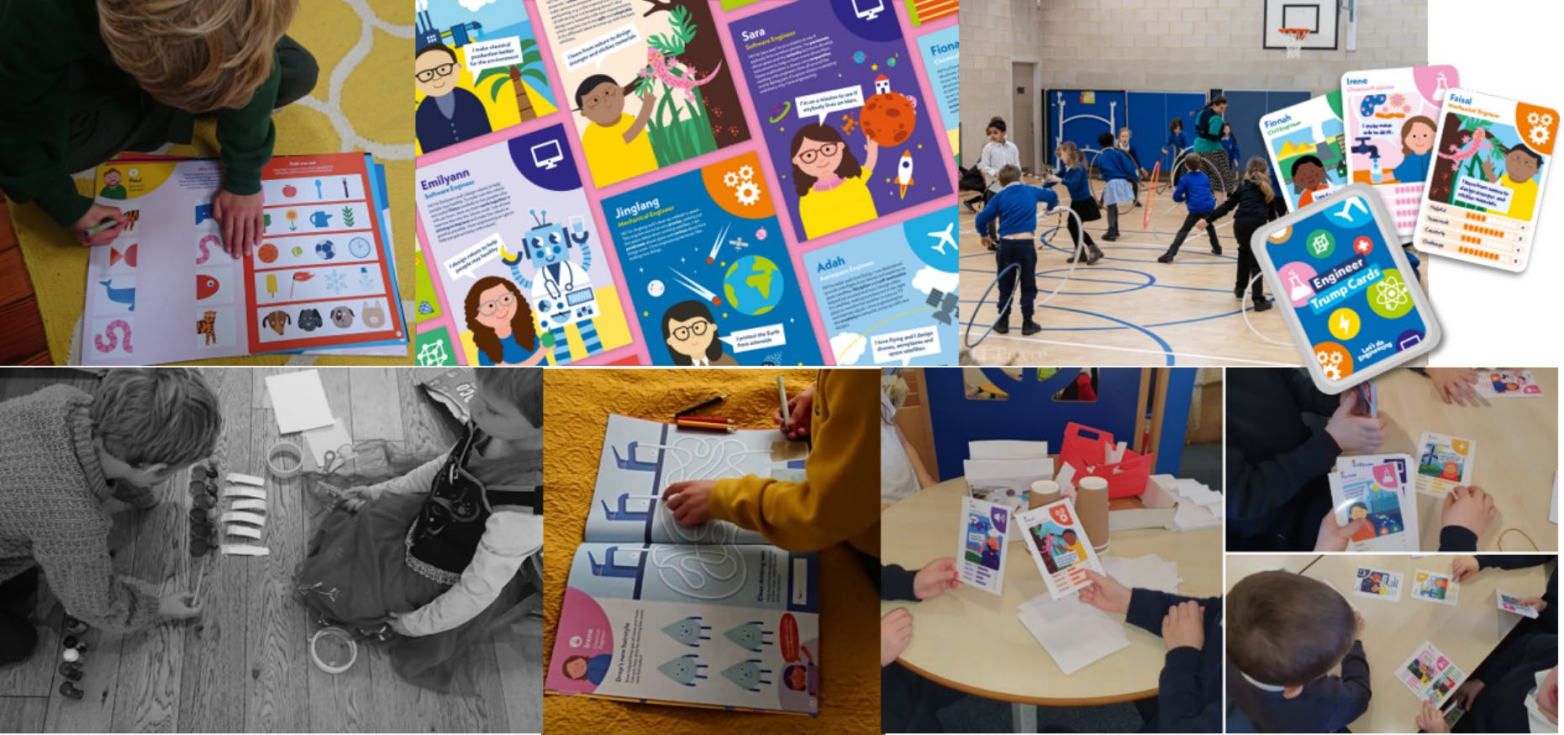
Let’s do engineering activities. From top left to bottom right: activity book being used; engineer classroom poster examples; circus engineering activities in action; engineer trump cards example; biomedical engineering activity example; different page of the activity book being used; top trumps game in action at a school

Over 4000 children received Let’s Do Engineering activity books, alongside the delivery of one or more Let’s Do Engineering activities within their schools and nurseries (Fig. 1). Several teachers took part in training sessions before embarking on activities and resources, with positive feedback around boosting teacher confidence and expanding engineering knowledge:

- “The engineering workshop was fascinating. It opened my eyes to how we can incorporate engineering ideas into our practice. We will benefit from the links made to gender inequality and Developing the Young Workforce (Scottish career development programme)” Teacher 1
- “The engineering training was fantastic, and the resources make it a lot less scary to introduce engineering into class” Teacher 2
- “My view of what an engineer is has changed” Nursery practitioner 1

Initially, teachers were given free choice to select their own activities which, in some cases, resulted in limited uptake, possibly due to lack of time to read through and investigate all of the different activities. Specific guidance on which activities and resources to implement was generally appreciated. Feedback on the Let’s do Engineering project, and the implementation of specific resources was requested, initially via an online survey to be completed after each activity. The numbers of educators completing the survey was low and so emails and phone calls were utilised instead.

“The website is very user friendly and easy to navigate. A one-stop-shop for everything, I was impressed with it and films were very child-friendly” Teacher 3

“All children have been really engaged, especially the girls.” Nursery practitioner 2

“We’ve only just started but from the questions and discussions it is having an impact already” Teacher 4

“A key positive is how the activities are linking different areas of the curriculum, great that they combine learning areas” Teacher 5

The expressive arts activities, e.g., drama, dance, circus, and song, received positive feedback with teachers reporting high levels of engagement. One nursery found the songs were really useful for building links with families, with families intrigued to find out more about the songs they were hearing the children sing at home. Educators reported adapting and building on the resources, making minor changes to suit their circumstances, e.g. putting out similar or linked toys to encourage further play.

Comparison of feedback on the same activity delivered in different settings revealed differences in the way that the experiences were scaffolded and supported by the adults, and how this worked to support learning. One setting felt that the ‘build a tower’ activity, which explores the stability of structures on different foundations, was enjoyable and engaging for the children but that they struggled to draw conclusions from their experiences. Another setting n which the same activity was implemented in a slightly different way, describing much more adult questioning to support the decision making and reflections of the children throughout the process was much more successful. One reason might have been teacher experience (Lippard et al., 2017). This could be an interesting future area of research to explore pedagogical approaches and the impact of training on teacher efficacy and the benefit of the resources.

### Teacher Viewpoints (RQ3 and RQ4)

Survey data showed that none of the educators in the focus group had grown up in a household with an engineer, although the majority now knew at least one scientist and engineer within their families and friends. Their general perceptions were that their friends and family were interested in science and engineering, with over 60% of them taking part in science conversations at least once a month; the corresponding figure for engineering was 30%. Additionally, while they felt that both science and engineering were useful to everyday life, the percentage of strongly agree responses was higher for science. Overall, the participants had a relatively high level of interest in science and engineering and a desire to find ways to engage learners in their settings with science and engineering, as has been previously noted in the literature (Kurup et al., 2019; MacDonald et al., 2021; Wan et al., 2021). In terms of barriers to implementing engineering with children under seven, the main issue was a lack of appropriate resources, followed by a lack of confidence, a lack of time and finally a lack of knowledge about engineering and engineering pedagogies, again as previously found (Bogue et al., 2012; Mackay et al., 2020; Van Aalderen-Smeets et al., 2012). However, all of the educators were able to name various activities which they were already undertaking. Examples included child-led ideas for the provision of particular materials (blocks, construction materials, loose parts) and junk modelling, to more formal approaches like the use of engineering habits of mind and design cycles or STEM challenges or subscription-based curriculums with progressive sequences of lessons.

Focus group findings are summarised below, highlighting the key factors which influence the resources and activities that teachers are interested in utilising. First, creative, hands-on active approaches to learning, especially songs, were appreciated by the teachers, with one group comparing Let’s do Engineering with their school's choice of subscription curriculum, which they felt was too heavy in its use of worksheets. On the other hand, they felt that the Let's do Engineering Meet the Engineer videos were engaging and they indicated they could see how they would work in class. Despite focus group participants not having been directly asked to implement activities, some had already started using the videos in class. It was also noted that having sign language made the videos inclusive.

“Our current materials are worksheet heavy and watching video heavy. When the kids hear STEM, they are really excited to go blow things up. What we have now doesn’t do that so it’s good to have something to supplement that.” Teacher 6

“I’m into expressive arts so the coral ones and the ones that start with song and the ones that start with movement are really attractive to me.” Teacher 7

“The videos in meet the engineers were a good length, and loved the follow on activities.” Teacher 3

“The videos were good and very usable. They were easy to use and interesting and interesting to read about the stereotypes.” Teacher 8

Second, linking STEM to the broader curriculum was viewed as a key element to emphasise, particularly through the use of stories and books.

“I’d never really thought about adding books and being able to put them out in provision is great.” Teacher 9

“Fantastic that the lessons start off with a story as a hook, great that they combine learning areas” Teacher 10

“I would sell it to staff as a one-stop-shop to lots of different areas rather than just STEM. The biggest things are transferable skills for the children in the world of work. You’ll be missing a trick with not linking the literacy and numeracy skills to what is needed in science and engineering.” Teacher 6

“The fact that it is mapped to Curriculum of Excellence [Scottish national curriculum] is really good. STEM is sometimes feared as an additional thing.” Teacher 11

Third, cost of materials and staff support are major considerations in selecting teaching resources. Activities need to be self-directed, particularly with ratios of 1 teacher to 29 children and little help from teaching assistants.

“We were really impressed with how user friendly the activities were” (but) “There were a few that came with a price tag due to cost of materials required” (and) “They’ve gone on the back burner [because] we would be the ones having to pay for it”. Teachers 6 and 9

“The easier it is to pick up and just make it work, the easier it is for teachers to do it. There is a lack of capacity with time and resources so the easier you can make it the more likely it is to be used.” Teacher 12

“I think there’s always the worry with practical activities is the staff. With younger children you need eyes in the back of your head. Planning to do a STEM activity when you’ve got maximum capacity with staff.” Nursery practitioner 3

There were however suggestions on how to implement certain resources and activities that might not be practical in a whole class setting. For example, in small rural settings (where school classes might comprise children from multiple age groups) or schools with ‘buddying’ systems, older children in composite classes may be able to support younger ones with activities. Additionally, building links with families via take-home activities was another suggestion.

“We had put things (activities) in the ‘no’ pile that we could see would be very beneficial for individual children and were talking about using them to put together as a lending library that could be sent home with the individual children. What’s nice is that you could give different packs to different children. Then you can do the ones in the class time that are more achievable to one big group or groups of children” Teacher 5

Finally, in the focus groups with preschool teachers, those following child-led pedagogies struggled to see how they might utilise the activities that were presented more like formal lesson plans. The Scottish national curriculum’s Early Years Framework (Scottish Government, 2009) outlines a play-based curriculum for 3–5 year olds, which includes children in their first year of primary school. Early years practitioners in Scotland often do not have planned lessons in the same way as teacher’s working in later years of primary school. There is no set time to do an activity—play is more free flow depending on the children. This means there is a lot of thinking on your feet and bringing out the resources as and when the children want. This play-based approach is shown to benefit learning (EEF, 2023) and links well with STEM learning (McClure et al., 2017) when teachers are able to integrate these perspectives. In this context, having materials to guide practitioners on how to ‘stage’ or introduce the activity in a child-led way is key and therefore it might be useful to provide advice on how to display the materials to encourage play-led learning as play provocations**.** One practitioner said she would stage the ‘build a tower’ materials and then chat about what they found out during milk/snack time.

## Discussion

Within the Let’s do Engineering study a wide range of activities and resources based around twenty engineer role models were co-created. Over thirty different activities were designed through pairings between engineer role models and creative practitioners, which incorporated music, dance and drama, circus, games, and arts and crafts, alongside engineering challenges. In total, at the time of writing, over 4000 children in Scotland had taken part in one or more activity and received a copy of the Let’s do Engineering activity book. This article has focused on the impact upon the participating engineer role models, creative practitioners and teachers. Here the findings (Table 3) and implications are discussed in relation to previous literature. Utilising the data from this evaluation, multiple recommendations have been generated for the implementation of similar projects, where scientists and engineers develop resources for use in schools and preschools, which are presented and discussed below.

**Table 3:** Linking research questions and findings for RQs 1–4

Research Question	Findings
What was the impact of the project on engineer role models and were they inspired to engage in further public engagement activities?	Increased knowledge and confidence to engage in public engagement with young children—overcoming one barrier identified in previous literature Reported a positive change in their approach to public engagement Inspired to become more involved in public engagement
How well did engineer role models and creative practitioners work together? What did they learn and what could be improved for future initiatives?	The networking opportunity was appreciated by both participants, i.e., having contacts for future collaborations and generating new ideas Creative practitioners reported frustrations with engineer’s timeframes, linking with previous literature showing that academics often feel they lack time for public engagement
How useful did teachers and nursery practitioners find the resources and activities?	Engineer role model videos were particularly appreciated Hands-on activities and expressive arts were popular Teachers reported seeing an impact on children Training given prior to resource use received positive feedback in increasing confidence. This links with the lack of educator confidence reported in the literature and statistics showing teacher confidence in engineering is lower than science (35% vs 70%) There were a lot of resources and it was hard for educators to screen and select what they needed
Which factors controlled whether particular resources or activities were used in various settings?	Pedagogy was a key factor, with settings that took a child-led approach struggling to implement activities presented as more of a formal lesson plan Links to the curriculum are helpful, especially achieving wider curriculum goals. This links with previous literature where teachers report a lack of time for STEM activities Linking with books was also a positive factor, and literature backs up the use of a story-led approach to engage children Low-cost and easy to source materials essential

Recommendations arising from each of these findings, which thus address RQ5, are presented in discussed in the “Discussion” section below

### Engineer Role Models and Creative Practitioners (RQ1, 2 and 5)

#### Impact on Participant Attitudes (RQ1)

Creative practitioners and engineer role models reported a positive impact on their confidence both to create content for this age group, and to communicate science and engineering. Lack of knowledge, skills and confidence, especially relating to pedagogies for young children (Bogue et al., 2012), has been noted in the literature (Mackay et al., 2020; Woitowich et al., 2022). Previous work has found that prior public engagement training, which has relatively low uptake and researchers can struggle to access (Weingart et al., 2021) and confidence (~ 50% of researchers self-reporting confidence) are not well-linked (Burchell, 2015). This study supports provides evidence of the value of ‘on-the-job’ training and learning from peers for early years educators.

#### Evaluation of the Engineer-Creative Practitioner Collaborations (RQ2)

In general, public perceptions of engineering are narrow, with many people not knowledgeable about what engineers actually do (RAEng, 2019). Discovering more about different engineering areas and how engineers work prompted the creative practitioners to reflect on the similarities with processes they use in their work and generated multiple ideas for collaborations. The engineer role models and creative practitioners both reflected that they now have a wider network to draw on to support future public engagement work, and that they would have appreciated further opportunities to interact within the project. Bringing together sciences and arts can generate tensions due to differing epistemologies and practices (Newman, 2023) and studies of interdisciplinary collaborations suggest extra time is required to build relationships (Bridle et al., 2013). Incorporating greater time for practitioners from different backgrounds to understand each other’s approaches and practices would be useful.

Previous work has found researchers need help to generate two-way engagement with the public (Hawke et al., 2020; Woitowich et al., 2022). Effective co-creation with children (Carballido et al., 2024; King-Kostelac et al., 2022) and teachers (Gelir, 2022; Gordon & Cullimore, 2023) has been previously described. This was limited in the current project due to Covid. Inviting teachers and schools into the project development stages and making broader collaborations (e.g. creative practitioners, engineer and teacher and/or children) could save time as teachers will already know what will work pedagogically, developmentally, cognitively, and within the daily routines, schedules, and resources of educational settings. However, this might open up other challenges, and lead to greater levels of uncertainty.

In this project, both creative practitioners and engineer role models wanted clearer parameters in terms of time, budget, expectation, opportunities for discursive interaction and collaborative problem solving, and testing. Creative practitioners and engineer role models were given an open brief in how they could work together. One respondent noted this was better for academic and creative minds. However, ambiguity around expectations and timeframes created some challenges and artists in particular reflected that tighter parameters, setting out expectations, roles, responsibilities, resources and timeframes, would have helped sustain the momentum in communications. Including teachers would add another degree of complexity in managing timeframes and expectations. Including children also requires careful consideration of multiple factors relating to power dynamics, methods of communication, and selection of co-creation approach (Hansen, n.d.).

#### Recommendations (RQ5)

Based on the above research findings and discussion three recommendations were generated to support improved co-creation between engineers and creative practitioners. First, create plenty of opportunities for engineers and creative practitioners to network and interact to generate, and develop, new ideas. Second, provide opportunities for ideas to be tested, and refined with end-users, allowing engineers and creative practitioners the opportunity to be involved in this process. Finally, an agreement detailing expectations, roles, responsibilities, resources and timeframes is essential to guide the collaboration.

### Educators (RQ3–5)

#### How Useful Did Educators Find the Resources? RQ3

Multiple studies suggest educator confidence in science and engineering is low (Kurup et al., 2019; MacDonald et al., 2021; Wan et al., 2021). Additionally, primary school teachers and nursery practitioners have similar narrow perceptions of engineers as the general public (RAEng, 2019), as illustrated by work with the Draw an Engineer studies undertaken with teachers (Vo & Hammack, 2021). One important factor, highlighted in both the focus groups and in feedback directly to the project lead, was the role of the Let’s do Engineering resources in enhancing teacher confidence by building and expanding their knowledge of what an engineer does. Given that one constraint on engineering education is teacher confidence (Wan et al., 2021), which could link with children’s inability to relate their science and engineering learning to careers (Padwick et al., 2022), broadening teacher perspectives on what engineers do could help overcome these challenges. Within the Van Aalderen-Smeets et al. (2012) model, this improvement in engineering understanding links with a reduced perceived difficulty and increased self-efficacy.

Literature suggests that other barriers for educators include a lack of appropriate resources (Leung, 2023), a lack of time (Jamil et al., 2018) and finally a lack of knowledge about engineering and engineering pedagogies (Bogue et al., 2012; Mackay et al., 2020). These findings were reflected in our work as well, with aspects of Let’s do Engineering being demonstrated to tackle some of these issues.

Creating links with the broader curriculum for engineering activities is critical to counter issues of a lack of time, and to highlight how engineering activities can also support key targets like literacy and numeracy. Educators in this study appreciated the cross-curricular nature of the resources, improving the perceived relevance of engineering (Van Aalderen-Smeets et al., 2012), by being able to link to existing provision in terms of storybooks, the transferrable skills which support a link to the world of work and the resources being curriculum mapped. The use of the expressive arts and play-based approaches reduced educator anxiety and increased enjoyment (Van Aalderen-Smeets et al., 2012).

Educators also highlighted that a lack of time to incorporate science and engineering can also extend to lack of time to screen, assess and select resources and suggested collating into a set of learning progressions or highlight curriculum links and learning outcomes early (or if online use filtering tools to enable this) to support selection of activities.

Challenges still remained around effective pedagogies with some educators reporting that they struggled to support their children to draw learning from some of the activities, whereas others felt the same activities worked well in their classroom. Professional development specifically linked to delivery of resources could support this point, further increasing teacher self-efficacy, and/or generating a way to enable educators to share their approaches. Furthermore, some educators made adaptions and additions to the resources. A and a way of sharing these adaptations would be useful for enabling peer-to-peer teacher support.

#### Which Factors Control Whether Particular Resources or Activities were Used in Various Settings? RQ4

Although previous studies have identified a lack of resources for early engineering acting as a barrier (Leung, 2023), these lack details relating to effective resource and activity design. Incorporation of role-models and counter-stereotypical imagery is reported to be beneficial (Aladé et al., 2022; Finnegan et al., 2015; Olsson & Martiny, 2018; Shimwell et al., 2023), and these aspects were also valued by the educators in this study, with multiple educators finding the role model videos useful. Other work has found storytelling approaches helpful for motivating problem-solving (Fleer, 2021), and in this study linking storybooks with the activities was also valued by educators.

Commonly used approaches in early years education focus on play-based activities, with the use of stories and songs being very common. However, these are little studied particularly in relation to songs and science (Na et al., 2014), although STEAM is growing in popularity (Su et al., 2024). Educators in this study valued the expressive arts approach utilised in some of the resources and enjoyed the hands-on nature of all the activities. Bringing together multiple subject areas, as in STEAM but also including literacy, expressive arts and curriculum goals such as introducing the world of work, was also valued.

Suggestions for improvements on specific activities included practical guidance on factors such as letter styles/fonts (e.g., using lower case letters written in the font children are usually shown in the activity book for children to trace and practice their writing), simplifying fine motor skills demands where possible (e.g., adapting the rocket building activity from one involving a lot of cutting and taping to a simpler build), including PowerPoint slides for the teacher to use on the big screen when introducing structures, or colour coding the drama instructions for ease of use. This illustrates the benefits of involving educators in resource development. Educators had various extension ideas and modifications for different activities, linking with other resources in the setting or other activities. Enabling teachers to share these ideas and their positive experiences could support others to enhance their practices.

Potential barriers to the uptake of resources are the cost and/or difficulty to source materials and the level of staff support required. Ideally, resources should be designed, and potentially co-created with educators, to ensure easy classroom implementation although other solutions such as buddying approaches with older children supporting younger ones or loan kits to families were suggested to enable young children to access all the resources developed in Let’s do Engineering. Educator confidence and experience with engineering pedagogies is also important (Lewis et al., 2021; MacDonald et al., 2023; O’Neill et al., 2023; Van Aalderen-Smeets et al., 2012) and there was a tension between the presentation of resources as quite detailed lesson plans and child-led pedagogies.

#### Recommendations (RQ5)

Based on the above research findings and discussion five recommendations were generated to support the improved design of early years engineering resources. First, ensure activities are low-cost; use resources that are easy to source and work effectively and safely in settings with larger groups of children. Second, make sure resources and activities are linked to the curriculum and save educators time by delivering on other key curriculum areas (e.g., literacy and numeracy) while also building STEM/engineering skills and knowledge; make it easy for educators to identify activities based on the curriculum links. Third, use STEM role models and counter-stereotypical imagery where possible and link this with a variety of activities, ideally using a story-led approach. Fourth, hands-on and creative activities are popular with educators, which may require engineers to collaborate with creative practitioners to access skills around music, dance, drama and other creative approaches. Finally, create opportunities to involve educators in resource and activity design at the earliest stage of creation, incorporating their feedback in initial design and throughout the development process.

Additionally, further recommendations relate to educator support and research. Firstly, it is important to provide training to educators about what engineering is and effective pedagogies to broaden perspectives and increase confidence. Secondly, there is a need for more research relating to the roles of adult-led and child-led activities in early years engineering, and STEM more broadly. This article also highlighted that the research field would benefit from further exploration into how best to introduce science and engineering topics when using child-led pedagogies (Skene et al., 2022). Educator attitudes and conceptions of their professional role in relation to preschool science learning has previously been shown to impact their pedagogical approach (Sundberg & Ottander, 2013). Here, clear differences in educator approach were noted in the feedback and could be explored more in observational studies, to identify pedagogical approaches which maximise learning in this context. This knowledge could then be utilised to develop further training resources, such as how-to videos, examples of questioning, and recommended resource set-ups, to support educators to maximise the benefit of the activities.

### Limitations

The study is based on the response of only half of the participating engineer role models and perhaps those who chose not to engage in feedback had different experiences and perspectives. Additionally, the teachers in the focus group appeared to have a relatively high level of science and engineering capital, perhaps because participation in the focus groups was voluntary and those interested in this topic chose to engage with us. Additionally, all but one of the respondents were female and white, with none reporting being disabled. Therefore, the findings are therefore not reflective of all practitioners, especially those with little knowledge of engineering or from different backgrounds. Our findings are also focused on the perspective of the adult’s involved. Research into the benefits of Let’s do Engineering for children will be the focus of future studies. Additionally, both engineer role models and creative practitioners reflected that they would have liked to test and refine their ideas with children, a factor that was limited due to the project running during the COVID-19 pandemic. Co-creation with teachers was also limited during COVID.

## Conclusion

Let’s do Engineering was developed to inspire young children and to provide practical and helpful resources for teachers. By focusing on providing resources for children at the beginning of their school career, Let's do Engineering is helping tackle the gender equality gap present in the engineering workforce by targeting children before career limiting decisions are taken. Highlighting the diversity of engineering by combining real-world engineer role models with creative approaches to teaching, Let’s do Engineering is a unique educational intervention that is much needed to support engineering learning in Scotland and beyond.

The aim of this practical article has been to share our findings with others setting out on similar collaborative journeys. From this evaluation, we have been able to highlight best practices for fostering collaborations between STEM professionals and creative practitioners. We have gained knowledge on what the educators of 3–7 year olds are looking for when searching for learning materials, and have identified factors that help or hinder the use of learning resources in classrooms.

## Supplementary Information

Below is the link to the electronic supplementary material.

Supplementary file1 (DOCX 18 KB)
