# Supplementary material for: Let’s Do Engineering: Engineers and Creative Practitioners Experiences of Co-creating Activities and Resources for 3–7 Year-Olds, and Teacher Evaluation of Resource Effectiveness
Source: Early Child Educ J. 2025 Mar 1;54(2):803–19. doi: 10.1007/s10643-025-01858-2 (PMC12904914; doi:10.1007/s10643-025-01858-2)
Supplement: Supplementary file 1 — Supplementary file1 (DOCX 18 KB) [file 10643_2025_1858_MOESM1_ESM.docx]

**Let’s Do Engineering: engineers and creative practitioners experiences of co-creating activities and resources for 3-7-year-olds, and teacher evaluation of resource effectiveness**

SUPPLEMENTARY INFORMATION

Example Questions

**Survey**

Which types of support did you access when you were developing your resource?

What other support would have been useful?

If you discussed the creation of your resource with a creative partner, please tell us more. E.g. did you meet more than once? How much time did you spend together?

What did you learn from working with a creative partner?

What were the challenges of working with a creative partner?

Have you developed any new working relationships or contacts as a result of this project? Tell us a little more about this.

Likert scales to rate aspects of the project, their previous experience in public engagement and the impact

**Interview Questions**

For engineers and creative practitioners: in developing resources what else would have been helpful? What went well? What could be improved?

Discussion areas: interest/motivation to participate; feedback on the process/approach; impact

For educators: which activities have you used? Do you have any suggestions for improvements? What can you tell us about the impact of the resources and activities on the children?

What else do we need to know about the effectiveness of the Let's Do Engineering design and support from your perspective?

**Requested Educator Preparation Prior to Focus Groups**

Survey:

Definitions of Engineering

Characteristics associated with engineers

Likert scale ratings on items such as “I know a lot about what engineers do” or “I am familiar with engineering ideas and concepts (suitable to discuss with 3-7 year olds)”

What kinds of science and engineering activities do you usually do with your children? Or how do you incorporate science and engineering learning at your setting? If planning science and engineering where do you look for ideas and inspiration?

What are barriers to implementing engineering activities within your setting?

PREPARATION:

Spend 5-10 mins exploring the Let's Do Engineering website - note down your initial thoughts and impressions.

Spend 10 mins looking through the different activity guides - sort them into those you'd be interested in doing with your class, those you might consider and those you don't think would be suitable. Make a few notes about why you've made these decisions.

**Focus Groups Agenda**

1. Introduction
2. Discussion of initial impressions of website

- What did you find out?
- What was appealing?
- What might have put you off?

1. Discussion of factors influencing activity/resource choice. What is most important to you when picking resources?

(Prompts: Curriculum relevance, availability of materials/resources, children’s interest, own prior learning, topic/theme set by school, staffing needed/available, confidence with resource/topic, easy to use).

1. Analysis of activities sent to the teachers:

Would like to do with your class
Would be nice to do but would need to adapt in some way
Would not like to do in with your class.

Identify the reasons why you categorised the activities into those groups (Consider factors identified at stage 3)

1. Planning - how would you use these activities in your setting? Develop a quick plan - identify any barriers
